# Molecular Dynamics to Elucidate the DNA-Binding Activity of AlpZ, a Member of the Gamma-Butyrolactone Receptor Family in *Streptomyces ambofaciens*

**DOI:** 10.3389/fmicb.2020.01255

**Published:** 2020-07-02

**Authors:** Cláudia M. Vicente, Jean-Michel Girardet, Laurence Hôtel, Bertrand Aigle

**Affiliations:** ^1^Université de Lorraine, INRAE, DynAMic, Nancy, France; ^2^Université de Lorraine, INRAE, IAM, Nancy, France

**Keywords:** molecular dynamics, DNA-binding, signaling molecule receptor, quorum sensing, kinamycin

## Abstract

Signaling molecule receptors play a central role in quorum sensing and in the coordination onset of specialized metabolite biosynthesis in *Streptomyces* due to their dual function in signal detection and gene expression control through DNA-binding in the promoter region of their target genes. In *Streptomyces ambofaciens* the *alp* biosynthetic gene cluster includes the signaling molecule receptor AlpZ that negatively regulates through a complex regulatory cascade the expression of key genes involved in the kinamycin antibiotic production until its cognate ligand, a yet unidentified signaling molecule, prompts its release from target promoters. Here we use an original molecular dynamics method to evaluate the DNA-binding properties of AlpZ to its target DNA sequence and the impact the signaling molecule has on the interaction. It is the first time this approach is used to characterize a regulator from the γ-butyrolactone receptor family. The observed K_D_ in the nanomolar range indicates that AlpZ-DNA constitute a particularly stable complex. The signaling molecule ably disturbs this binding while kinamycin has no effect on the activity of AlpZ. Regulator size was determined and found to be considerably large regarding protein sequence, indicating that AlpZ regulates gene expression by binding the DNA as a homodimer, and structural modeling comparison with closely related γ-butyrolactone receptors supports this conclusion.

## Introduction

Bacterial quorum sensing is a well-established phenomenon by which bacteria produce and sense chemical signals to communicate with neighboring cells. These diffusible signaling molecules are able to trigger a plethora of activities such as genetic exchange, antibiotic production, motility, virulence, biofilm formation, among others. The signaling molecule accumulates extracellularly during cell growth and when the concentration reaches a certain threshold it is detected by a receptor that elicits a downstream signal transduction cascade, triggering a specific gene expression program. An array of signaling molecules are used for cellular communication. For example, the acylhomoserine lactones (AHL) are the representatives and most studied chemical signals in Gram-negative bacteria, while Firmicutes mainly use peptides as signaling molecules (Waters and Bassler, 2005). *Streptomyces*, ubiquitous filamentous Gram-positive bacteria characterized for their complex life cycle, linear chromosome and their ability to produce a wide variety of specialized metabolites including antibiotics, herbicides, immunosuppresssants and anticancer agents, display a different profile of signaling molecules.

The vast majority of signaling molecules produced by Streptomycetes and identified to date are classified into three major groups, γ-butyrolactones (GBL) (Yamada et al., 1987; Ohnishi et al., 1999; Takano et al., 2000; Takano, 2006), 2-alkyl-4-hydroxymethylfuran-3-carboxylic acids (AHFCA) (Corre et al., 2008) and γ-butenolides (Kitani et al., 2011; Arakawa et al., 2012). Two other less studied signaling molecules with quite different structures exist, the PI factor and the N-methylphenylalanyl-dehydrobutyrine diketopiperazine (Recio et al., 2004; Matselyukh et al., 2015). These molecules are widespread in the genus, often controlling both specialized metabolism and morphological differentiation (McCormick and Flärdh, 2012). Many of these molecules are active at nanomolar concentrations.

Gamma-butyrolactones constitute the largest and most studied group of signaling molecules in *Streptomyces*, and the first bacterial signaling molecule to be discovered was the A-factor of *Streptomyces griseus* (Khokhlov et al., 1967), a γ-butyrolactone type signaling molecule that governs a vast regulon of genes involved in both the morphological differentiation and specialized metabolism (Horinouchi et al., 2001; Horinouchi, 2002). Specialized metabolite biosynthetic pathway expression is a much regulated process (Liu et al., 2013). The onset of production is tightly controlled by a regulatory network comprised of different transcriptional regulators incorporating various environmental and physiological signals (Bibb, 2005; van Wezel and McDowall, 2011; Martín and Liras, 2012). Cluster-situated regulators (CSRs) play an important role, directly controlling gene transcription within the specialized metabolite biosynthetic gene cluster [although some CSRs have been described to cross regulate other biosynthetic gene clusters (Vicente et al., 2015; McLean et al., 2019)], while pleiotropic regulators modulate the biosynthesis of several specialized metabolites and often morphological development as well. Some of these transcriptional regulators are central to the quorum sensing phenomenon, acting as receptors of the diffusible signaling molecules, and usually belong to the large and widely distributed TetR family of transcriptional regulators (Ramos et al., 2005). A typical TetR-family GABR (γ-butyrolactone receptor) is comprised of two functional domains, a N-terminal helix-turn-helix DNA-binding domain that can interact with specific DNA target sequences in promoter regions, and a C-terminal signaling molecule-binding domain that interacts with cognate ligands (Cuthbertson and Nodwell, 2013). The promoter-bound signaling molecule receptor prevents transcription, and binding of the cognate signaling molecule leads to its release from the DNA allowing gene expression (Willey and Gaskell, 2011; Sidda and Corre, 2012).

The type II polyketide synthase (PKS) *alp* biosynthetic gene cluster of *Streptomyces ambofaciens* is responsible for the production of the antibiotic kinamycin and its regulatory network has been previously elucidated (Pang et al., 2004; Aigle et al., 2005; Bunet et al., 2008, 2011). Several forms of kinamycin have been identified (Bunet et al., 2011) and for the purpose of this work will be referred to as kinamycin. The cluster is duplicated (two identical copies) due to its location in the terminal inverted repeat (TIR) sequences at both ends of the chromosome and was initially identified as comprising 27 genes including three genes that compose the minimal PKS (*alpA*, *alpB*, and *alpC*) and five regulatory genes. Recent reports indicate that the cluster size could be extended to include a 24 gene region containing 6 genes responsible for the synthesis of the diazo functional group (Wang et al., 2015). Of the regulator genes, *alpT*, *alpU*, and *alpV* are members of the SARP (*Streptomyces* antibiotic regulatory proteins) family (Wietzorrek and Bibb, 1997), and *alpW* and *alpZ* belong to pseudo-GABR and GABR family, respectively (Matsuno et al., 2004; Nishida et al., 2007). Pseudo-GABRs are paralogs of GABR but are unable to interact with the cognate GBL. In the regulatory cascade (Supplementary Figure S1), the positive regulator AlpV activates kinamycin biosynthesis promoting the expression of the minimal PKS-encoding genes. During the initial growth phase, the negative regulator AlpZ specifically binds and prevents expression of the operon *alpXW* and of *alpV* as well as its own, hindering antibiotic biosynthesis particularly through the repression of *alpV*. The binding sites of AlpZ, as well as those of AlpW, have been characterized (Bunet et al., 2008, 2011) and show high homology to previously defined ARE (autoregulatory elements) motifs (Folcher et al., 2001). Following synthesis and build-up of the yet unknown *S. ambofaciens* signaling molecule, it binds to the regulator AlpZ releasing it from the target promoters and allowing gene expression that leads to kinamycin production (Bunet et al., 2008). At this stage late negative regulator AlpW accumulates and blocks *alpV* expression thus switching off kinamycin biosynthesis, at which point the signaling molecule concentration also decreases and based on transcription analysis data it is hypothesized that the negative control exerted by AlpZ on the expression of both *alpV* and *alpXW* resumes (Bunet et al., 2008, 2011). Although it remains unidentified, the signaling molecule responsible for the quorum sensing regulation of kinamycin production in *S. ambofaciens* has been characterized to some extent. Initial studies predict that it is not a γ-butyrolactone type signaling molecule, based on its physical properties such as its resistance to alkaline conditions and also to high temperatures (Bunet et al., 2008), but rather perhaps a AHFCA-like molecule. Furthermore, an *afsA*-like gene responsible for its biosynthesis was identified close to the *alp* cluster (unpublished data). AfsA-like enzymes have been shown to be key in both γ-butyrolactones and AHFCA synthesis (Hsiao et al., 2007; Kato et al., 2007; Corre et al., 2008).

As in the case of kinamycin biosynthesis, these signaling molecules and their receptors constitute one of the most extended regulatory systems to elicit the biosynthesis of specialized metabolites and/or morphological differentiation in a coordinated fashion in *Streptomyces* (Polkade et al., 2016). Several signaling molecules have been identified so far with more expected to exist (Thao et al., 2017). For this reason, signaling molecule receptors are central to modulate specialized metabolite biosynthesis and can be used as a strategy for cryptic gene cluster activation (Aigle and Corre, 2012; Sidda and Corre, 2012), shaping regulatory systems and to develop expression control tools (Bowyer et al., 2017; Biarnes-Carrera et al., 2018).

In this study, we address the role of AlpZ, a negative regulator and receptor of an unknown signaling molecule in *S. ambofaciens* that controls kinamycin biosynthesis, using a novel biophysical-based approach that allows a detailed depiction of the regulator’s DNA-binding activity and how it is affected by the signaling molecule.

## Results

### Regulator AlpZ Has High Affinity to Its Target Sequence

**FIGURE 1:**
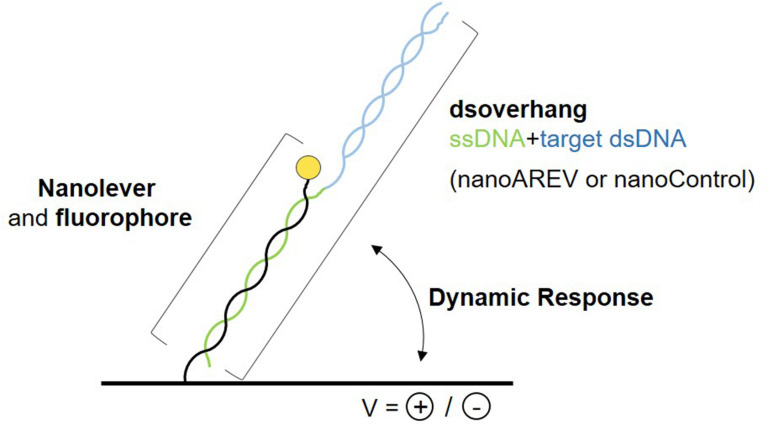
Schematic representation of the molecular dynamics system. The dsoverhangs, the nanoAREV and nanoControl, include a dsDNA region containing either the protein’s model target DNA or an unspecific DNA region, respectively (blue), and a ssDNA sequence (green) that is complementary to the tethered nanolever (black), which has a fluorophore linked at its end (yellow circle). Alternating the electric potentials applied to the gold microchip surface will either attract or repel the negatively charged dsDNA. The distance to the surface affects the intensity of the fluorescent light emitted by the dye due to the quenching effect of the gold surface, allowing the accurate measurement of the oscillating orientation change of the DNA in dynamic response.

A recently developed technology was used to perform molecular dynamics studies and characterize the putative signaling molecule receptor AlpZ, the switchSENSE^®^. It is based on the electric properties of negatively charged double stranded DNA sequences, that when grafted to a gold microelectrode and subjected to alternating electric potentials are either attracted or repelled from the surface. Real-time tracking of the fluorescence emission from the fluorescent dye attached to the dsDNA nanolevers that depends on its proximity to the gold surface, allows to assess variations in the switching movement. To determine the affinity of AlpZ to its target sequences through molecular dynamics, specific DNA sequences were designed, the dsoverhangs. These contain both the target dsDNA sequence and a ssDNA region complementary to the biochip attached nanolevers (Figure 1). The AREV sequence located in the promoter region of the *alpV* gene (i.e., AREV) was used as a model of the known recognized sequences in the gene cluster (Bunet et al., 2008) and to create the dsoverhang nanoAREV. Functionalization of the dsoverhangs on the biochip was verified before each assay through increased fluorescence signal detection.

**FIGURE 2:**
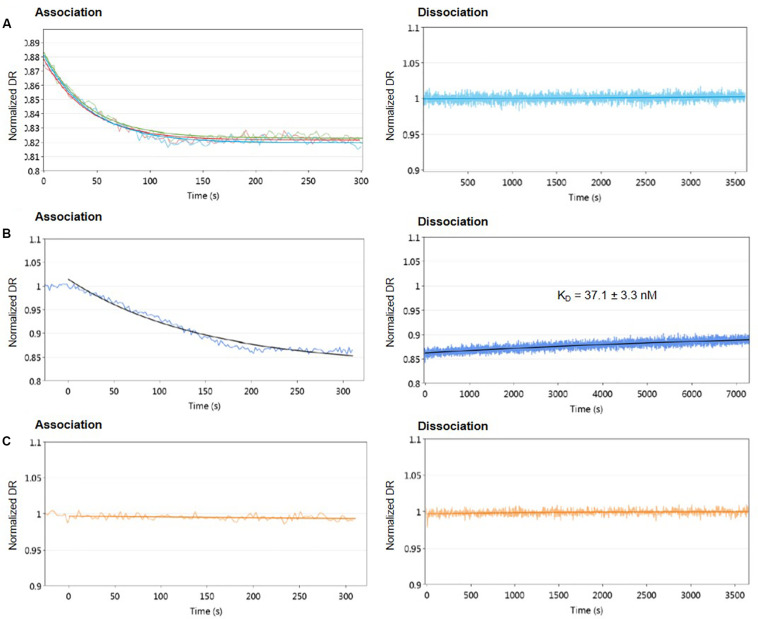
Real-time measurements of molecular dynamics of AlpZ using molecular dynamics. Both association and dissociation analysis were performed. Different AlpZ protein concentrations were used with the nanoAREV dsoverhang. Colored lines in green, light blue and red represent the protein concentrations 55.6 μM, 5 μM, and 1.6 μM, respectively **(A)**. Assays were also carried out with 50 nM of AlpZ and nanoAREV dsoverhang **(B)**, and with 50 nM AlpZ and nanoControl dsoverhang **(C)**. Dissociation constant (K_D_) shows the mean and standard deviation of three independent assays. Signal expressed as normalized dynamic response (DR).

First, different concentrations of protein from 1.6 to 55.6 μM were used to assess the DNA-binding activity of AlpZ to the nanoAREV. Results show a strong affinity and remarkable stable binding of AlpZ to its target sequence. Indeed, in these conditions binding rate constant *k*_ON_ values reached 1.46 to 4.43 × 10^4^ M^–1^.s^–1^ (Table 1), respectively. Notably, with increasingly higher protein concentrations the *k*_ON_ remains unchanged suggesting a saturation phenomenon occurs easily (Figure 2A left panel, Table 1). Furthermore, in these conditions no dissociation was observed even after a long period of time in dissociation favoring conditions (Figure 2A right panel). To better analyze the binding properties of AlpZ to the nanoAREV, assays were then performed using a lower concentration of protein (50 nM). It was observed that in these conditions protein binding occurs more slowly, with a *k*_ON_ of 1.38 × 10^5^ M^–1^.s^–1^ (Figure 2B left panel, Table 1). Additionally, a slow dissociation is finally observed, although an even longer period of time is necessary to reach a steady state. This dissociation is however, enough to determine the binding rate constant *k*_OFF_, which is of 1.05 × 10^–4^ s^–1^. The dissociation constant K_D_ of AlpZ to its target model sequence was found to be 37.1 nM (Figure 2B right panel, Table 1). These results indicate that the AlpZ-DNA complex is substantially stable.

**TABLE 1:** Kinetic parameters of AlpZ with target DNA sequence.

Concentration (M)	*k*_ON_ (M^–1^.s^–1^)	*k*_OFF_ (s^–1^)	K_D_ (nM)
55.6 × 10^–6^	4.43 ± 0.16 × 10^4^	N/A	N/A
5.0 × 10^–6^	4.68 ± 0.16 × 10^4^	N/A	N/A
1.7 × 10^–6^	1.46 ± 0.05 × 10^4^	N/A	N/A
50.0 × 10^–9^	1.38 ± 0.06 × 10^5^	1.05 ± 0.08 × 10^–4^	37.1 ± 3.3

To ensure AlpZ specificity in binding DNA during the assays, an unspecific dsoverhang was designed, the nanoControl. Instead of the AREV sequence it contains the AREU sequence originally located in the promoter region of *alpU* from the kinamycin gene cluster. It has been previously demonstrated that this sequence, which contains a weakly conserved ARE motif, is neither recognized nor bound by the regulator (Bunet et al., 2008). Molecular dynamic analysis using the established conditions and the nanoControl indicate that AlpZ does not bind to the dsoverhang. There is no observed association and consequently no dissociation (Figure 2C), confirming that AlpZ recognizes and specifically binds its target DNA sequences.

### Sizing and Protein Structure Modeling Suggest AlpZ Functions as a Homodimer

**FIGURE 3:**
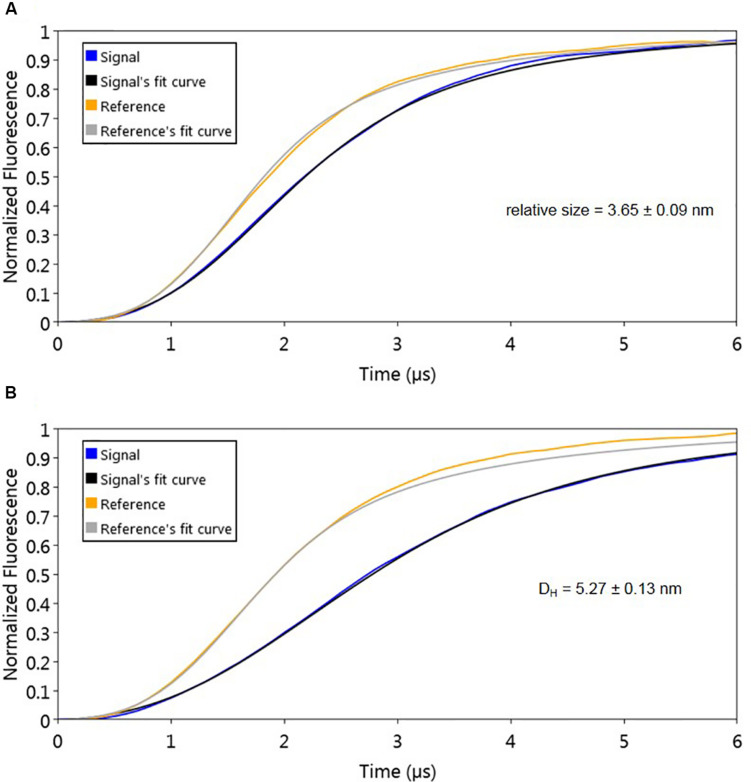
Protein size analysis. Hydrodynamic diameter (D_H_) was determined by comparing size of the nanoAREV dsoverhang **(A)** with that of dsoverhang nanoAREV + AlpZ complex **(B)**. Results show the mean and standard deviation using at least 3 independent assays. Signal expressed in normalized fluorescence.

The used molecular dynamics approach enables the measurement of protein friction, ultimately allowing protein size determination through hydrodynamic diameter (D_H_) measurements. As the experimental set-up uses dsoverhangs, the movements of the biochip’s nanolevers with the dsoverhangs used for the analysis have increased friction and lower dynamic response (DR) compared to those of the nanolevers alone. It is therefore necessary to first determine the relative size of the dsoverhang (different from the actual D_H_ as the dsoverhangs do not have a globular shape that is assumed in the model for size calculation). By comparing the switching dynamics of the dsoverhang to those of the reference nanolevers, the nanoAREV was shown to have 3.65 nm in relative size (Figure 3A), identical to the size of the nanoControl, as expected since the dsovehangs have the same length (data not shown).

**FIGURE 4:**
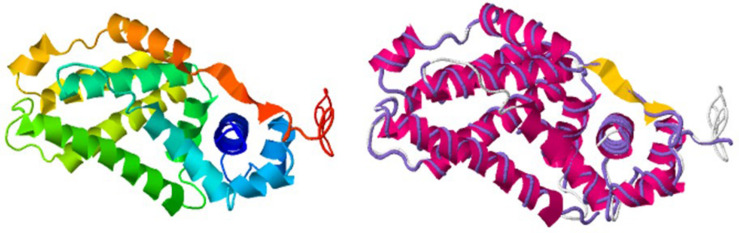
AlpZ structure prediction. Predicted model of AlpZ **(left)** and alignment with TylP, a TetR regulator of tylosin biosynthesis in *S. fradiae* (**right**, structure analog in purple).

Interestingly, we determined that AlpZ has a D_H_ of 5.27 nm (Figure 3B). AlpZ is a relatively small protein with 237 aa and a theoretical MW of 25.7 kDa, however, this result suggests its size is considerable. The only other related γ-butyrolactone receptor with structural data available is CprB from *Streptomyces coelicolor* A3(2) (Natsume et al., 2004). AlpZ and CprB belong to the GABR and pseudo-GABR family, respectively and show significant similarity (almost 30% identity and CprB has 26.4 kDa, and moreover similar predicted structures). Although it has been demonstrated that CprB binds DNA as a dimer of dimers (Bhukya et al., 2014), further biophysical modeling analysis using the switchANALYSIS software and the structural data of the apo-form of CprB (Natsume et al., 2004) predicted its D_H_ to be 5.21 nm, a very similar size to that of AlpZ. This result indicates that the form of AlpZ that binds the nanoAREV could be a homodimer. Furthermore, using a threading/fold recognition method (Zhang, 2009; Yang and Zhang, 2015) to modelize AlpZ it was possible to predict its single molecule structure. The best model has a relatively good C-score of −0.60 and is structurally very close to the homodimer structure of the TetR family regulator TylP from *Streptomyces fradiae* (Ray et al., 2017), showing a TM-score of 0.87 and coverage of 0.822 (Figure 4). This result further strengths the hypothesis that AlpZ is active as a homodimer.

### Signaling Molecule Drastically Disrupts AlpZ-DNA Complex

**FIGURE 5:**
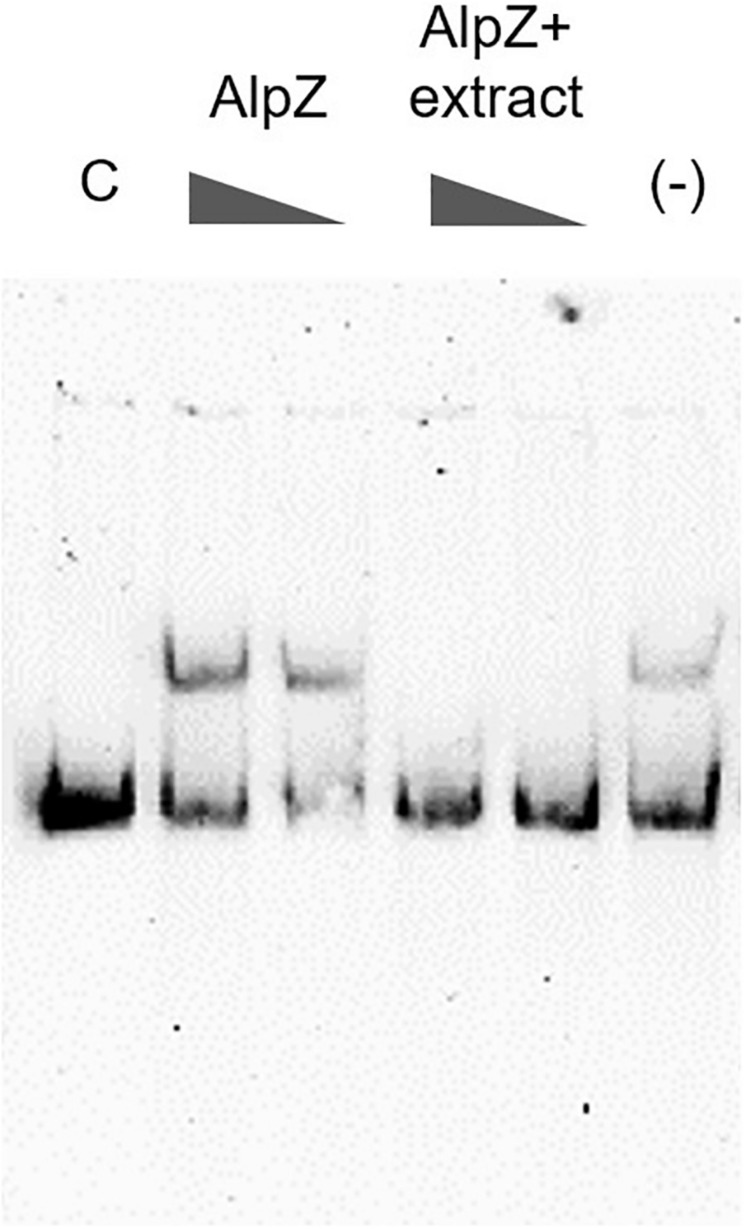
Analysis of the presence of signaling molecule in mutant strain extracts. Electrophoretic mobility shift assays (EMSA) were used to assess AlpZ protein DNA-binding activity to a probe comprising the AREV sequence, with conditions of decreasing protein concentration (160 and 80 nM, respectively) and decreasing amounts of a *S. ambofaciens* ΔΔ*alpW*ΔΔ*alpID* strain supernatant extract containing the signaling molecule (25 μg/ml and 2.5 μg/ml, respectively) using 80 nM of protein. The loss of shifted bands indicates the presence of signaling molecule. Lane *C* contains the control without protein and lane *(-)* methanol 50%.

It has been previously reported that a yet unidentified *S. ambofaciens* signaling molecule is able to affect the DNA binding by AlpZ, effectively modulating the regulation of kinamycin biosynthesis (Bunet et al., 2008). To obtain signaling molecule containing extracts, the *S. ambofaciens* ΔΔ*alpW*ΔΔ*alpID* mutant strain was used as it lacks the late regulator *alpW* leading a constitutive production of signaling molecule (unpublished data). This strain is also deficient in kinamycin production [loci *alpIABCD* deleted, in which the *alpABC* locus encodes the minimal PKS (Pang et al., 2004)], allowing therefore analysis without the putative interference of the biosynthetic pathway products (i.e., kinamycins or its intermediates). Extracts obtained from the culture supernatant were routinely checked for the presence of signaling molecule using electrophoretic mobility shift assays (EMSA), as seen in Figure 5.

**FIGURE 6:**
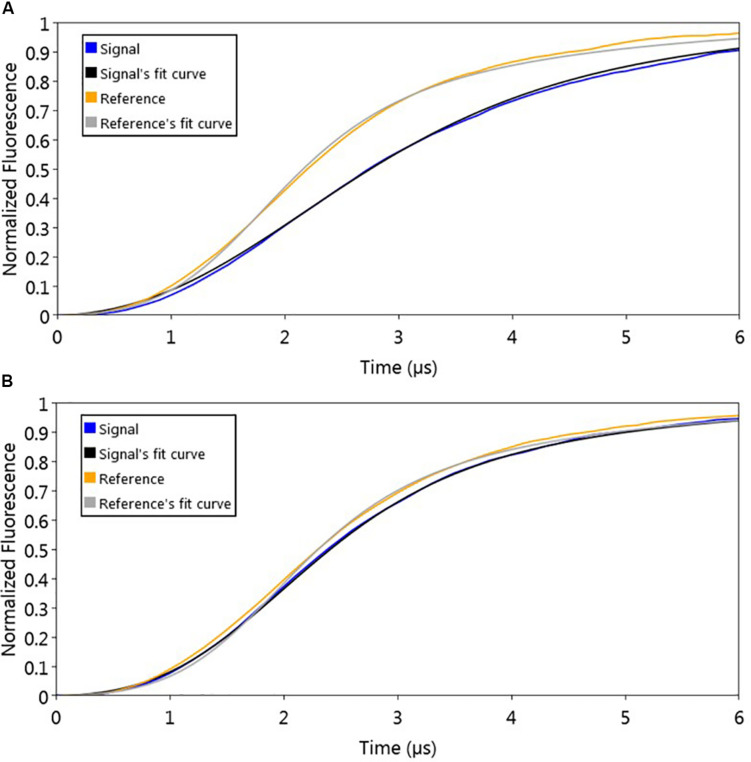
Sizing analysis used to determine signaling molecule effect on AlpZ DNA-binding activity. **(A)** Analysis following preparation step with AlpZ binding to nanoAREV and **(B)** after adding signaling-molecule containing extract (0,5 mg/ml). Signal expressed in normalized fluorescence.

**FIGURE 7:**
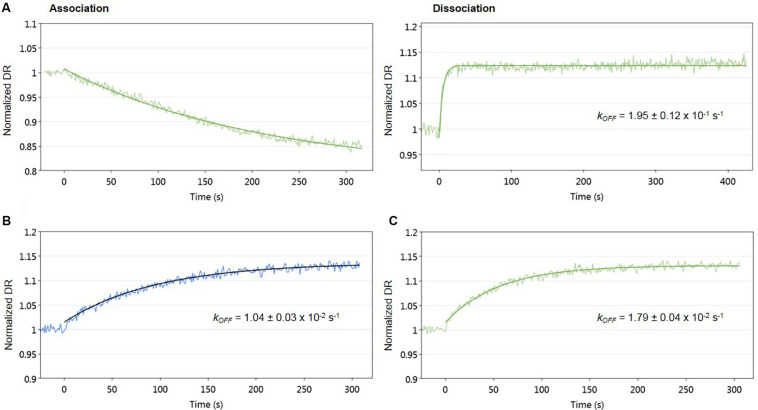
Impact of signaling molecule on AlpZ binding activity. Kinetic analysis of AlpZ binding to the nanoAREV and its release in presence of concentrated signaling molecule containing extract **(A)** and protein dissociation with 25 μg/ml **(B)** and 50 μg/ml **(C)** of signaling molecule containing extract. Signal expressed as normalized dynamic response (DR).

To evaluate the effect of signaling molecule on AlpZ and its binding to the nanoAREV, an assay was designed to accurately detect protein release from the dsoverhang. The correct binding of AlpZ to the dsoverhang is first verified, and then an extract containing signaling molecule is added and molecular dynamics analysis are performed. Size analysis confirmed that the signaling molecule drastically alters the DNA binding function of AlpZ. The protein that was steadily bound to the dsoverhang (Figure 6A) shows a size of 5.7 nm, and is released when signaling molecule is present, changing the detected size to that of the dsoverhang and reference with 3.5 nm (Figure 6B). Additionally, kinetics analysis also demonstrates that the impact of the signaling molecule can be significant. Concentrated extracts of 0.5 mg/ml induce an almost immediate release, showing a considerably high *k*_OFF_ of 1.95 × 10^–1^ s^–1^ (Figure 7A), whereas more diluted samples lead to a fast but steady dissociation event with no protein molecule remaining bound to the dsoverhangs after just 5 minutes (Figures 7B,C). The observed effect is manifestly concentration dependent, and even small modifications of concentration affect the *k*_OFF_ values. Extract samples from the ΔΔ*alpW*ΔΔ*alpID* strain at 25 and 50 μg/ml display *k*_OFF_ values of 1.04 × 10^–2^ and 1.79 × 10^–2^ s^–1^, respectively. Other solutions were previously proven to have no effect on the DNA-binding activity of AlpZ, including the used culture medium and purified molecules of the three subclasses of γ-butyrolactones, the A-factor, SCB1 and IM-2 (Bunet et al., 2008), confirming the observed phenomenon is specifically related to the unknown *S. ambofaciens* signaling molecule.

**FIGURE 8:**
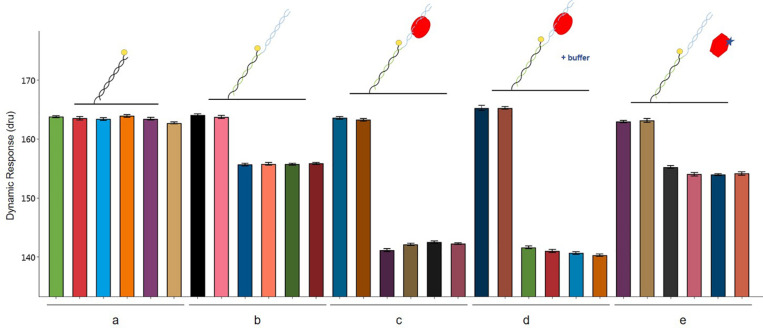
Conformation analysis of AlpZ binding activity. Comparing changes in dynamic response of nanolevers alone **(a)**, after annealing of the dsoverhangs **(b)**, following binding of 50 nM of AlpZ **(c)**, in presence of PE40 buffer **(d)**, and signaling molecule-containing extract **(e)**. Above the graph are representations of the assay conditions and kinetics on the nanolevers, nanoAREV dsoverhang, with AlpZ (red circles), in the presence of buffer (+buffer) and signaling molecule (star). Vertical bars show dynamic response in the 6 electrodes present in the biochip. In each case the first two electrodes are the controls with nanolevers to verify the absence of unspecific binding to this region, and the next four electrodes were subjected to the different conditions. Assay was performed with 50 nM of AlpZ and a concentrated signaling molecule containing extract (0.5 mg/ml). Signal expressed in dynamic response units (dru). Error bars represent the standard deviation.

The interactions involving the three players AlpZ, target DNA and signaling molecule are clearly visible with a conformational analysis. The assay was designed and performed to analyze AlpZ binding to the nanoAREV dsoverhang and subsequently adding PE40 buffer (control) and signaling molecule containing extract to observe its effects on the association, followed by a conformational analysis (Figure 8). As in every performed assay, six electrodes are used, the first two of which are the controls without neither ligand nor analytes, and the following four electrodes are used for the assay itself (Figure 8a). As expected, the functionalization of the dsoverhangs (the first step of the assay), already has a negative impact on the dynamic response (Figure 8b). When AlpZ is introduced the dynamic response further decreases, as the protein tightly binds to the dsoverhangs (Figure 8c). The complex protein-DNA is very stable and not altered by solutions like the buffer PE40 (Figure 8d). However, when in the presence of signaling molecule containing extracts the dynamic response increases to the original levels upon release of the dsoverhangs by AlpZ (Figure 8e). During the entire assay, the control electrodes remain unchanged, confirming that no unspecific protein binding occurs to the nanolevers.

### Kinamycin Does Not Impact AlpZ DNA-Binding Activity

**FIGURE 9:**
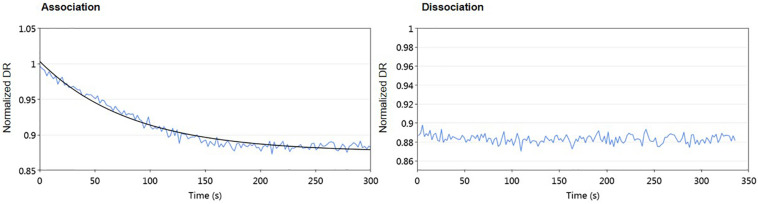
Kinamycin has no effect on DNA-binding activity of AlpZ. A solution of pure kinamycin D at 50 μg/ml was used. Association analysis from AlpZ binding to nanoAREV in preparation for the assay **(left panel)**, and dissociation analysis after adding kinamycin D **(right panel)**.

Some regulator-receptors have been demonstrated to bind the product of their biosynthetic gene clusters in *Streptomyces*, such as JadR2 (Xu et al., 2010). To analyze if kinamycin could also act as a ligand of AlpZ, we performed molecular dynamic assays in the presence of purified kinamycin D and the results were analyzed (Figure 9). No dissociation of AlpZ was observed when kinamycin was used, confirming the regulator does not respond to kinamycin as a signal to modulate transcriptional control. Although the *S. ambofaciens* signaling molecule remains elusive, these results support the initially proposed classification of AlpZ as a true GABR.

## Discussion

The TetR regulator AlpZ plays a key role in the regulatory network of kinamycin biosynthesis in *S. ambofaciens* ATCC 23877, coupling it to the production of a yet unknown signaling molecule. As a negative transcriptional regulator, it exerts its control by binding specific promoter located sequences and hence blocking gene expression. An in-depth molecular characterization of this regulator is crucial to understand how it responses to target promoters and triggering signals.

The present study indicates that AlpZ has a strong affinity for its target DNA sequences and forms very stable complexes upon binding, as seen by a K_D_ in the nanomolar range. This result is also in good agreement with the EMSA experiments performed. Signaling molecule receptors such as GABRs have long been known for their affinity to DNA targets (Miyake et al., 1989; Onaka et al., 1995), though recent characterization of the binding activity of CprB to its target DNA shows a considerably higher K_D_ in the micromolar range (Biswas et al., 2015). The stable AlpZ-DNA complex explains how the regulator achieves a steady control on gene expression and on subsequence kinamycin biosynthesis. Protein size was determined through molecular dynamics, an approach validated in previous studies by comparing it with other more recurrent methods (Blocquel et al., 2017). The determined size of DNA-bound AlpZ is hence consistent and nonetheless unexpectedly large, suggesting that the regulator could be in a homodimer form. A model of AlpZ structure obtained through a threading/fold recognition prediction method shows high similarities with that of TylP, also a homodimer (Ray et al., 2017), strengthening the hypothesis that AlpZ binds its target DNA as an homodimer.

Only one trigger has been identified to actively interfere with the AlpZ-DNA complex, the yet unidentified *S. ambofaciens* signaling molecule. When present, the signaling molecule prompts a rapid AlpZ dissociation and the slightest amount variation is detected and impacts the dissociation rate. Consistent data were found both with molecular dynamics and sizing assays, substantiating this conclusion.

It is unclear if the DNA release is a result of an interference mechanism (e.g., competition for the same binding site), or rather the induction of structural conformational changes on the regulator hindering its DNA-binding capability. Based on previous studies, the latter hypothesis seems more probable, as TetR regulators appear to shift between mutually exclusive conformational states, either binding DNA or their cognate ligand of choice (Ramos et al., 2005; Bhukya et al., 2014), unlike the LuxR receptor regulator in proteobacteria whose structure has been resolved in complex with both the target DNA and the cognate pheromone (Zhang et al., 2002). Some bacterial compounds have been described to function as both antibiotics and signaling molecules (Beyersmann et al., 2017), however, that does not seem to be the case of kinamycin in regard to AlpZ. The DNA-binding activity of AlpZ is not impacted by kinamycin, indicating the regulator is not able to bind the antibiotic in contrast with other regulators that have been described to bind intermediates or products of their gene cluster, such as TylP (Ray et al., 2017), MphR(A) regulator of erythromycin biosynthesis (Zheng et al., 2009), SimR in *Streptomyces antibioticus* (Le et al., 2009) and the regulator of tetracyclin production TetR (Hinrichs et al., 1994). However, we hypothesize that one of the other regulators present in the *alp* cluster, AlpW, could perhaps be able to bind kinamycin or one of its biosynthetic pathway intermediates, as it has been described to be the case for other pseudo-GABRs, such as JadR2 and jadomycin (Xu et al., 2010).

The interactions between AlpZ-DNA complex and signaling molecule constitute a remarkably sensible system for gene regulation and induction of kinamycin biosynthesis, where even small concentration changes are sensed and impact protein-DNA complex stability. The promoter region of the *afsA*-like gene identified in the proximity of the *alp* cluster has been shown to contain an ARE motif (unpublished). It would be interesting to explore the role of AlpZ in the expression regulation of the *afsA*-like gene. One could envision that the AlpZ-ARE*afs*A-like complex would activate gene expression for a positive feed-back loop of induction of production of signaling molecule. Even though the nature of this signaling molecule continues to elude us, this work clearly demonstrates that the response of AlpZ to the extract of *S. ambofaciens* is in no way linked to kinamycin and broadens the knowledge on AlpZ regulatory role, possibly paving the way for similar autoregulator receptors. Efforts are underway for the purification and identification of the *S. ambofaciens* signaling molecule. The discovery of new signaling molecules in *Streptomyces* is impaired by the fact that these compounds are typically produced in very small amounts. Moreover, purification scale can be highly variable, avenolide in *S. avermitilis* required 2000 L of fermentation broth whereas *S. coelicolor* furans were identified using only 40 squared Petri dishes (Corre et al., 2008; Kitani et al., 2011). Approaches being used include the traditional large-scale fermentation method and specific capture using GABRs (Yang et al., 2005; Zou et al., 2014).

Although several studies exist on autoregulator receptors, there is still a long way to go to identify the cognate ligands of all TetR regulators identified to date (Cuthbertson and Nodwell, 2013). The present work constitutes the first described study of a signaling molecule receptor using an innovative molecular dynamics approach and provides a further step on the way to identifying the signaling molecule in *S. ambofaciens*. Furthermore, understanding how these regulators control gene expression and respond to the trigger of signaling molecules will ultimately contribute to the development of new approaches for specialized metabolite discovery and production improvement, as well as advancing other application tools.

## Materials and Methods

### Strains, Media, and Growth Conditions

Strains used in this study are listed in Table 2. *Streptomyces* strains were grown in R2 medium (Kieser et al., 2000) and *Bacillus subtilis* was grown in LB. Kinamycin production was assessed on R2 medium as described previously (Pang et al., 2004; Aigle et al., 2005).

**TABLE 2:** Bacterial strains and cosmids/plasmids used in this work.

Strain	Description	References
*S. ambofaciens* ATCC 23877	Wild type	Pinnert-Sindico, 1954
*S. ambofaciens* ΔΔ*alpW*	*alpW* loci replaced by a scar	Bunet et al., 2011
*S. ambofaciens* ΔΔ*alpW*ΔΔ*alpID*	*alpW* and *alpIABCD* loci replaced by a scar	This work
F6ΔΔ*alpID*:scar	genomic library cosmid F6 with locus *alpID* replaced by a scar	Pang et al., 2004
*Escherichia coli* ET12567	strain used for interspecific conjugation	MacNeil, 1988
pUZ8002	for in *trans* mobilization of *oriT* containing cosmid	Paget et al., 1999

### Construction of the Mutant Strain ΔΔ*alpW*ΔΔ*alpID*

To obtain a strain of *S. ambofaciens* producing the signaling molecule controlling the biosynthesis of kinamycin but unable to synthetize the antibiotic itself, we first made an in-frame deletion in the ΔΔ*alpW* mutant strain (Bunet et al., 2011) of the locus *alpID* on both chromosomal arms. This locus includes the *alpA*, *alpB* and *alpC* genes encoding the minimal PKS and was replaced by a scar. The strategy was based on the REDIRECT system (Gust et al., 2003) and was carried out as described previously (Pang et al., 2004; Bunet et al., 2008). Only the start and stop codons of *alpI* and *alpD*, respectively remained after deletion and gene replacement was confirmed by Southern blot and PCR analysis using the CK1 and CK2 primer pair (Supplementary Table S1, data not shown).

### Extraction of AlpZ Signaling Molecule

To study the effect of *S. ambofaciens* signaling molecule on AlpZ DNA-binding crude extracts were prepared as follows. The mutant strain ΔΔ*alpW*ΔΔ*alpID* was grown in 50 ml R2 medium in 250 ml flasks and incubated at 30°C until late exponential-stationary transition phase. Supernatant was collected and extracted twice with 1 volume of ethyl acetate, dried in a rotavapor, and dissolved in 100 μl methanol-H_2_O 1:1 and stored at −20°C.

### DNA-Protein Binding Assays

DNA binding assays were performed by EMSA as described previously (Bunet et al., 2008). Briefly, AREV DNA probe was obtained by PCR using the primers listed in Supplementary Table S1 and directly labeled with digoxigenin using the DIG Oligonucleotide 3’-End Labeling kit, 2^nd^ Generation (Roche Applied Science). Binding assays were performed with AlpZ protein, purified as described previously (Bunet et al., 2008). Labeled probes (0.4 ng) were incubated at 30°C for 10 min with AlpZ protein (80–160 nM) in binding buffer (20 mM HEPES pH7.6, 1 mM EDTA, 10 mM (NH_4_)_2_SO_4_, 1 mM DTT, 0.2% Tween 20, 30 mM KCl) containing 50 μg/ml poly(dI-C) in a 20 μl final volume. When necessary, *S. ambofaciens* ΔΔ*alpW*ΔΔ*alpID* extracts (1 μl) were added after the incubation period and incubated for further 10 min to verify the presence of signaling molecule. Binding reactions were analyzed with 5% native PAGE and run in 0.5X TBE buffer. DNA was then transferred onto a positively charged nylon membrane (Amersham Hybon-N^+^) by electroblotting, then fixed by UV crosslinking, detected with anti-digoxigenin antibodies, and developed by chemiluminescence with the CDP-StarTM reagent (Roche Applied Science).

### Binding Kinetics and Hydrodynamic Diameter Measurements

A switchSENSE^®^ DRX instrument and MPC-48-2-R1-S biochips (Dynamic Biosensors GmbH, Martinsried, Germany) were used to characterize the binding kinetics and protein size changes (Knezevic et al., 2012; Langer et al., 2013).

Specific dsDNA sequences coupled with ssDNA overhang regions complementary to the ssDNA sequences tethered to the biochip (nanolevers) called dsoverhangs, were designed. To prepare the dsoverhangs 2 μM of the two comprising ssDNA sequences (listed in Supplementary Table S1) were annealed by heating at 95°C for 10 min and slowly cooled to room temperature in PE40 buffer pH 7.4 (10 mM Na_2_HPO_4_/NaH_2_PO_4_, 40 mM NaCl, 0.05% Tween20, 50 μM EDTA, 50 μM EGTA). For the analysis of protein affinity to the dsoverhang, 150 μl of AlpZ at a range of concentrations were injected with a flow rate of 30 μl/min, and dissociation was measured with running buffer at a flow rate of 30 μl/min over 120 min to 4 h. Assays to assess the effect of signaling molecule on the binding of AlpZ to DNA started with a preparation step with dsoverhang (the ligand, either nanoAREV or nanoControl) and 150 μl of AlpZ (the analyte) at 50nM with a flow rate of 30 μl/min without dissociation, followed by a sandwich type of capture injecting 140 μl of signaling molecule containing extract (second analyte) at different concentrations (dilutions performed in PE40) with a flow rate of 20 μl/min. All assays used dsoverhangs at 500 nM and were performed at 30°C, combining both association/dissociation and size measurements and used PE40 as running buffer. A regeneration solution (alkaline solution pH 13) was used to remove the ligand after each assay.

Binding rates constants (*k*_ON_ and *k*_OFF_) and dissociation constants (K_D_) were determined using real-time measurements of the switching dynamics. Protein size was estimated by comparing the switching dynamics of bound protein with those of bare DNA and with the lollipop biophysical model (Langer et al., 2013). Analysis was performed with the switchANALYSIS software from Dynamic Biosensors and using at least three independent assays.

### Protein Structure Modeling

Structure prediction was performed using a threading/fold recognition method and the server I-TASSER (Zhang, 2009; Yang and Zhang, 2015). After predicting secondary and solvent accessibility, structure templates with the highest significance in threading alignments are identified with LOMETS (Wu and Zhang, 2007). Structure models are predicted and ranked based on the C-score, a confidence score to estimate the quality of models predicted by I-TASSER. It is calculated based on the significance of threading template alignments and the convergence parameters of the structure assembly simulations, and values vary in a range of [−5, 2] where models with high confidence show higher C-score values (Zhang, 2008; Yang et al., 2015).

### Kinamycin D Purification

A solution of kinamycin D was purified by semipreparative HPLC on an Agilent 1100 instrument equipped with an Agilent Zorbax Eclipse RP-C18 column (21 × 100 mm, 5 μm) monitoring the absorbance at 420 nm as described previously (Bunet et al., 2011).

## Data Availability Statement

All datasets generated for this study are included in the article/Supplementary Material.

## Author Contributions

CV and LH performed the experiments. CV, J-MG, and BA analyzed the data. CV and BA conceived the experiments and wrote the manuscript. BA supervised the project and obtained the funding. All authors have read and approved the final manuscript.

## Conflict of Interest

The authors declare that the research was conducted in the absence of any commercial or financial relationships that could be construed as a potential conflict of interest.
