# Supplementary material for: Molecular Dynamics to Elucidate the DNA-Binding Activity of AlpZ, a Member of the Gamma-Butyrolactone Receptor Family in Streptomyces ambofaciens
Source: Front Microbiol. 2020 Jul 2;11:1255. doi: 10.3389/fmicb.2020.01255 (PMC7343708; doi:10.3389/fmicb.2020.01255)
Supplement: Supplementary file 1 [file Data_Sheet_1.docx]

Supplementary Material

# Supplementary Figures and Tables

## Supplementary Figures


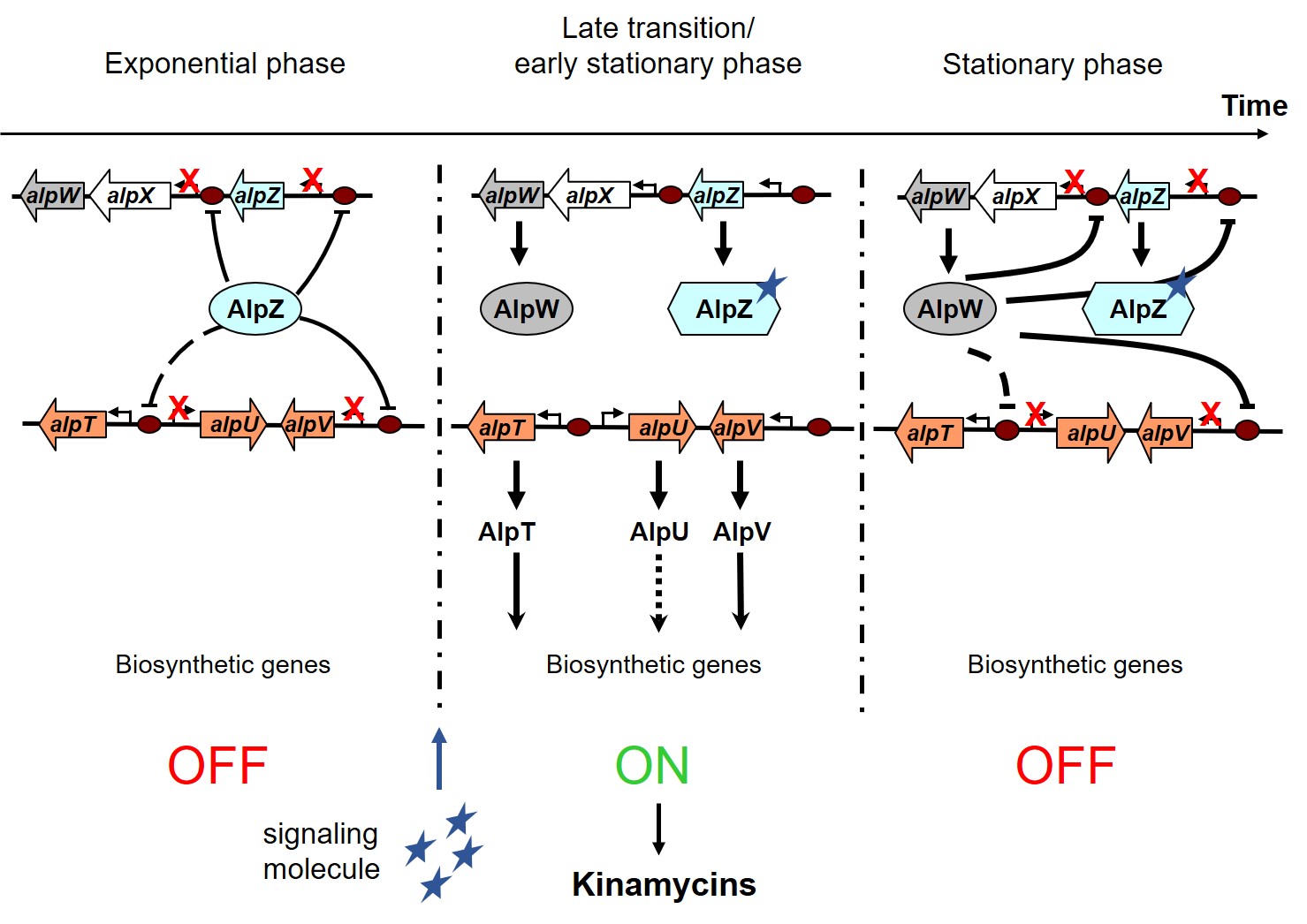


**Figure S1.** **Model of the regulation of kinamycin biosynthesis in *S. ambofaciens*.** In the initial growth phase, AlpZ represses the expression of the operon *alpXW*, the positive regulator encoding gene *alpV* and its own encoding gene by specifically binding the AREV sequences located in the promoter region (brown circles), preventing the expression of the biosynthetic genes and therefore leading to no kinamycin production. Then, the unknown signaling molecule accumulates and interacts during the transition phase with AlpZ releasing it from its binding sites, allowing gene expression and consequent kinamycin biosynthesis in the late transition to early stationary phase. In the later growth stages, the negative regulator AlpW represses expression of its targets *alpZ*, *alpXW* and *alpV*, effectively switching off kinamcyin biosynthesis. Note that the ARE motif located in the intergenic *alpT*-*alpU* region is not a target of AlpZ or AlpW, although deletion of either *alpZ* or *alpW* genes affects the expression of these SARP encoding genes.

## Supplementary Tables

Table S1. Primers used in this work

| Primer description and name | Sequence (5’-3’) |
| --- | --- |
| *Mutant strain verification* |  |
| CK1 | TGGTGGGCGGGAAGATGC |
| CK2 | CGAAGGGCGTGCGGCTCC |
| *EMSA probe* | |
| AREV-1 | GCCGTATCCGCCGCATCT |
| AREV-2 | CCTGACGCGGTTTGGGAG |
| *dsoverhangs* | |
| nanoAREV1 | CTCACATTGACAAACCGACTGTGCTGTTTTTTTATCAGGGATCAGCGTTCGATGCTTCCGACTAATCAGCCATATCAGCTTACGACTA |
| nanoAREV2 | CCCTGATAAAAAAACAGCACAGTCGGTTTGTCAATGTGAG |
| nanoControl1 | GGGGGGTGTGAGAACGGGGTGTTCTGGTGTTTGCGCCGGAATCAGCGTTCGATGCTTCCGACTAATCAGCCATATCAGCTTACGACTA |
| nanoControl2 | TCCGGCGCAAACACCAGAACACCCCGTTCTCACACCCCCC |
